# m6A Methyltransferase METTL3 Regulates Inflammatory and Immune Microenvironments in Renal Cell Carcinoma via Modulation of the PI3K/AKT Pathway

**DOI:** 10.1155/ijog/4975062

**Published:** 2026-04-29

**Authors:** Tao Cheng, Weiqiang Xu, Zeyu Zha, Likai Mao, Jing Ning, Shengtong Wang, Xinjie Huang, Mingli Gu

**Affiliations:** ^1^ Department of Urology, The Second Affiliated Hospital of Bengbu Medical University, Bengbu, 233000, Anhui, China

**Keywords:** immune microenvironment, METTL3, PI3K/AKT pathway, renal clear cell carcinoma

## Abstract

**Objective:**

The involvement of methyltransferase‐like 3 (METTL3, an m6A methyltransferase) and the PI3K/AKT pathway in tumor progression and immune regulation remains poorly understood. This study investigates whether METTL3 influences the immune microenvironment of renal cell carcinoma (RCC) via m6A‐dependent PI3K/AKT pathway activation and explores its translational potential.

**Methods:**

METTL3 expression was assessed in tumor and adjacent tissues from 34 RCC patients using Western blot and immunohistochemistry. The clinical cohort comprised 24 male and 10 female patients with an age of 61–77 years. METTL3 was genetically silenced or overexpressed in 786‐O cells, and the PI3K/AKT pathway was pharmacologically activated with 1,3‐dicaffeoylquinic acid (1,3‐diCQA, a PI3K/AKT pathway activator). Cellular proliferation (EdU assay), invasion (Transwell), and apoptosis (flow cytometry) were evaluated. Flow cytometry was employed to quantify PD‐L1, HLA‐I expression, and CD8+ T cell activity. Subcutaneous tumor xenografts in C57BL/6 nude mice were used to assess tumor growth and immune marker expression. METTL3, PI3K, and AKT co‐localization was examined via immunofluorescence.

**Results:**

METTL3 was elevated in RCC tissues (*p* < 0.05). Silencing METTL3 suppressed proliferation and invasion and promoted apoptosis in 786‐O cells (*p* < 0.05), concomitant with reduced PI3K/AKT pathway phosphorylation (p‐PI3K/PI3K, p‐AKT/AKT). Conversely, METTL3 overexpression or PI3K/AKT pathway activation (via 1,3‐diCQA) enhanced cell viability (*p* < 0.05). High METTL3 expression or PI3K/AKT pathway activation increased PD‐L1 (*p* < 0.05), decreased HLA‐I (*p* < 0.05), and impaired CD8^+^ T cell function (*p* < 0.05), whereas METTL3 knockdown reversed these immune‐evasion effects. Bioinformatic analysis further showed a positive association between METTL3 expression and neutrophil infiltration in TCGA‐KIRC, supporting a link between METTL3 upregulation and an inflammatory, immunosuppressive microenvironment. In vivo, METTL3 knockdown significantly attenuated subcutaneous tumor growth in nude mice (*p* < 0.05) and reduced PD‐L1 and CD163 expression (*p* < 0.05). However, cells pretreated with 1,3‐diCQA prior to inoculation counteracted the tumor‐suppressive effects of METTL3 silencing. Together, these findings support an m6A‐dependent METTL3–YTHDF1–PI3K/AKT pathway regulatory axis in RCC, although direct m6A modification of individual PI3K/AKT pathway transcripts was not examined in the present study.

**Conclusion:**

METTL3 drives immune evasion and promotes RCC growth by activating the PI3K/AKT pathway through an m6A‐dependent mechanism involving YTHDF1.

**Highlights**
1. METTL3 is upregulated in RCC and promotes tumor proliferation/invasion via PI3K/AKT pathway activation.
2. METTL3 modulates RCC immune evasion by regulating PD‐L1, HLA‐I, and CD8^+^ T‐cell function.
3. Targeting the METTL3–PI3K/AKT pathway axis may improve RCC immunotherapy efficacy.
4. METTL3 silencing attenuates RCC growth and reverses immunosuppressive crosstalk between RCC cells and CD8+ T cells (in vitro).

## 1. Introduction

At present, renal cell carcinoma (RCC) has developed widespread resistance to conventional radiotherapy and chemotherapy [1]. Immune checkpoint inhibitors (ICIs) are a new type of cancer treatment, but therapeutic resistance and significant interindividual variability in treatment response remain substantial clinical challenges [2]. Emerging evidence from RCC clinical samples and preclinical models indicates that TIME heterogeneity—particularly the balance between effector T cells and immunosuppressive subsets (e.g., Tregs, M2 macrophages)—plays a pivotal role in determining ICI response [3, 4]. The TIME comprises diverse immune cell populations, cytokines, and immunosuppressive signaling molecules, with its dynamic equilibrium directly governing immune evasion mechanisms and tumor progression [5]. Consequently, elucidating the regulatory mechanisms underlying the immune microenvironment in RCC and developing targeted strategies to reprogram this immunosuppressive niche hold profound implications for optimizing immunotherapeutic approaches. Recent evidence suggests that inflammatory programs within the RCC microenvironment, including neutrophil‐ and myeloid‐associated cytokine signaling, contribute to T‐cell dysfunction, immune escape, and reduced responsiveness to immune checkpoint blockade [6, 7]. These observations indicate that the inflammatory microenvironment is not merely a background feature of RCC but an active determinant of immunotherapy resistance.

In recent years, the critical role of epigenetic modifications in tumorigenesis and cancer progression has attracted considerable research interest. Among various epigenetic mechanisms, ribonucleic acid (RNA) methylation modifications, particularly N6‐methyladenosine (m6A), have emerged as crucial post‐transcriptional regulators. These modifications contribute to tumor cell proliferation, invasion, and immune evasion by modulating multiple aspects of RNA metabolism [8, 9]. Methyltransferase‐like 3 (METTL3) is overexpressed in solid tumors, including RCC, and its high expression correlates with poor prognosis by promoting tumor proliferation [10–12]. However, the precise involvement of METTL3 in reshaping the immune microenvironment of RCC and its molecular mechanisms remain poorly understood. A sentence has been added to state the specific knowledge gap more explicitly, namely that the coordinated role of METTL3 and PI3K/AKT pathway in regulating the RCC immune microenvironment remains insufficiently defined. Notably, the PI3K/AKT pathway, a master regulator of tumor metabolism and survival, not only drives malignant transformation but may also exert indirect effects on the composition and function of the TIME by secreting immunosuppressive factors or modulating immune checkpoint molecules [13]. Preliminary research indicates that METTL3 may regulate the PI3K/AKT pathway activity in an m6A‐dependent fashion [14]. Yet, whether this METTL3–m6A–PI3K/AKT pathway axis mediates immune microenvironment reprogramming in RCC awaits further investigation.

The present study will investigate the role of the METTL3–m6A–PI3K/AKT pathway regulatory axis in the RCC immune microenvironment. Specifically, we aim to (1) elucidate whether METTL3 modulates PI3K/AKT pathway activation through m6A modification and (2) determine how this regulatory axis affects tumor cell‐immune cell interactions. The findings may establish METTL3 as a promising therapeutic target to improve immunotherapy efficacy in RCC, while providing a scientific foundation for developing novel combination therapies based on epigenetic regulation mechanisms.

## 2. Materials and Methods

### 2.1. Study Population

Thirty‐four RCC patients were enrolled in the study (January 2024 and January 2025). The clinical cohort comprised 24 male and 10 female patients with an age of 61–77 years. These patients were pathologically diagnosed with RCC in our hospital. Presence of concurrent malignancies; diagnosis of immunodeficiency disorders; and active infectious diseases at the time of sample collection were excluded.

Paired tissue samples, including cancerous lesions and adjacent normal tissues, were obtained from all enrolled patients for subsequent analysis. The study was approved by the ethical committee of Bengbu Medical College (Approval number: 2024‐No. 163), and all participants provided written informed consent.

### 2.2. Detection of METTL3 Expression

#### 2.2.1. Western Blot

Tissue was lysed by RIPA to extract the supernatant, and protein samples were separated by SDS–PAGE and transferred to PVDF membranes. After blocking with 5% skim milk, the primary antibody METTL3 (1:1000, ab240595) and the internal control GAPDH (1:5000, ab9484) were added. The next day, HRP‐labeled goat antirabbit IgG secondary antibody (1:500) was added, visualized by ECL. Protein bands were quantified using ImageJ software (Version 1.8.0), with relative expression calculated as the gray value of the target protein [METTL3, p‐PI3K (ab154598), PI3K (ab139307), p‐AKT (ab324747), AKT (ab133458)] normalized to GAPDH. All antibodies were purchased from Abcam (USA); molecular weight markers were included in each gel to confirm protein size (METTL3: 70 kDa, PI3K: 127 kDa, p‐PI3K: 127 kDa, AKT: 56 kDa, p‐AKT: 56 kDa, GAPDH: 36 kDa). All Western blot experiments were independently repeated three times.

#### 2.2.2. Immunohistochemistry (IHC)

After being dewaxed, hydrated, and antigen repaired in citrate buffer, the paraffin tissue sections were blocked with 5% BSA and added with primary antibody against METTL3 (1:200). An HRP‐labeled secondary antibody (1:200) was added the next day, followed by DAB coloration and counterstained with hematoxylin. The slides were sealed with neutral gum and observed under a microscope. IHC positivity rate was quantified by two independent pathologists (blinded to group allocation) using the following criteria: 5 high‐power fields (400×) were randomly selected per section, and the number of positive cells (brown‐stained cells) was counted and divided by the total number of cells in each field. The average value of five fields was used as the positivity rate for each sample. Interobserver agreement between the two pathologists was evaluated using Cohen’s kappa coefficient (*κ* = 0.877), indicating substantial agreement. Discrepancies between pathologists were resolved by consensus.

### 2.3. Biological Information Analysis

The expression profiles (ENSG00000165819) were mapped to GeneSymbol, and the expression of METTL3 in tumor diseases and its association with prognosis were evaluated using the R package, and the correlation between the degree of immune infiltration and immune cells of METTL3 renal tumors was subsequently assessed according to IOBR.

### 2.4. Cell Grouping and Treatment

The human RCC cell line 786‐O cells (purchased from Henan Engineering Technology Research Center of Industrial Microorganisms, BNCC338472) were transfected with METTL3‐silencing constructs (Silence‐METTL3), METTL3‐overexpressing constructs (Elevated‐METTL3), or an empty pcDNA3.1 vector (rescue control for the second transfection) control (blank). 786‐O cells were cultured in RPMI‐1640 medium supplemented with 10% fetal bovine serum and 1% penicillin–streptomycin at 37°C in a humidified incubator containing 5% CO_2_. After 48 h of transfection, the cells were harvested for subsequent experiments. All gene constructs for abnormal METTL3 expression were designed and synthesized by Shanghai OBiO Technology Co., Ltd. Prior to transfection, 786‐O cells were plated in 6‐well plates at a density of 1 × 10^6^ cells per well. The culture medium was replaced with fresh medium containing 1,3‐Dicaffeoylquinic acid (1,3‐diCQA, purchased from MCE, HY‐N1412; CAS No.: 19870‐46‐3), was used as a PI3K/AKT pathway activator at a final concentration of 10 μM, dissolved in dimethyl sulfoxide (DMSO). This concentration was selected based on previous studies [15]. The vehicle control group (blank and Silence‐METTL3 cells) was treated with an equal volume of DMSO (final concentration ≤ 0.1%), which was verified to have no effect on 786‐O cell viability or PI3K/AKT pathway activity in preliminary tests, and the cells were incubated. Additionally, 786‐O cells were treated with a combination of Silence‐METTL3 and 1,3‐diCQA (first transfected with Silence‐METTL3, then treated with 1,3‐diCQA).

### 2.5. Validation of Intervention Effects

To verify the intervention effects, we performed Western blot analysis to examine the protein expression levels of METTL3, p‐PI3K (1:1000 dilution), PI3K (1:1000), AKT (1:1000), and p‐AKT (1:1000) in each experimental group, using the same methodology as previously described.

### 2.6. Measurement of Global m6A Levels

To quantify the global m6A methylation levels in 786‐O cells under different treatments, we performed an m6A RNA methylation assay using the EpiQuik m6A RNA Methylation Quantification Kit. For each sample, 200 ng of purified RNA was bound to a strip well precoated with an m6A capture antibody. The relative m6A level was calculated as a percentage of total RNA based on a standard curve and normalized to the blank control group.

### 2.7. METTL3 Enzyme Activity Dependence Assay

To validate the m6A‐dependent mechanism of METTL3, we constructed wild‐type (WT) and catalytic‐dead mutant (D395A)‐METTL3 expression vectors. The full‐length human METTL3 cDNA was cloned into a pcDNA3.1 vector (forward 5′‐GAG​TTC​GTG​GCG​GCC​TAC​GCC‐3′ and reverse 5′‐GTA​GCC​GTA​ATC​GGT​ACC​G‐3′). For rescue experiments, 786‐O cells were first transfected with Silence‐METTL3 using Lipofectamine 3000. After 24 h, cells were retransfected with either WT‐METTL3, D395A‐METTL3, or empty vector (2 μg per well in 6‐well plates). Cells were harvested 48 h post‐transfection for Western blot analysis of the PI3K/AKT pathway.

### 2.8. YTHDF1 Knockdown Experiment

A validated YTHDF1‐targeting siRNA (5′‐GCA​UGU​ACU​UCA​GCA​ACA​ATT‐3′) and a nontargeting scramble siRNA (control) were designed and synthesized by Shanghai GenePharma Co., Ltd. After transfection into cells, the expression of the PI3K/AKT pathway was detected.

### 2.9. EdU Staining

Cells were seeded in 24‐well plates at a density of 5 × 10^4^ cells per well. Following adhesion, the culture medium was replaced with fresh medium containing 10 μM EdU, and cells were incubated at 37°C for 2 h. Cells were fixed with 4% paraformaldehyde for 15 min and permeabilized with 0.5% Triton X‐100 for 10 min. For nuclear counterstaining, cells were incubated with DAPI for 5 min. All EdU assays were independently performed three times.

### 2.10. Transwell Migration Assay

786‐O cells were resuspended in serum‐free medium at a density of 2 × 10^5^ cells/mL, and 200 μL of the cell suspension was inoculated in the upper chamber, and complete medium containing 10% FBS was added to the lower chamber. After 24 h of incubation, the transmembrane cells were fixed and examined under a microscope. All Transwell assays were independently performed three times.

### 2.11. Flow Cytometry Analysis

After washing with PBS, the cells of each group were resuspended in the corresponding buffer for subsequent detection.

#### 2.11.1. Apoptosis

Cells were resuspended at a density of 1 × 10^6^ cells/mL in 1× binding buffer, 5 μL each of Annexin V‐FITC and PI were added, and incubated at room temperature in the dark for 15 min. The BD FACSCanto II flow cytometer was used to collect data.

#### 2.11.2. PD‐L1 and HLA‐I

The cells were resuspended in flow staining buffer containing 2% fetal bovine serum, and then anti‐PD‐L1‐APC and anti‐HLA‐I‐FITC antibodies were added, respectively. The cells were incubated at 4°C in the dark for 30 min, and the percentage of positive cells was detected after washing.

#### 2.11.3. CD8^+^ T Cells

CD8^+^ T cells were isolated from human peripheral blood mononuclear cells (PBMCs) by magnetic beads and co‐cultured with 786‐O cells at a ratio of 1:5 (the 1:5 ratio refers to CD8+ T cells:tumor cells). 786‐O cells were not irradiated and were not treated with mitomycin‐C before co‐culture in RPMI‐1640 medium containing 10% fetal bovine serum and IL‐2 (50 U/mL). CD8^+^ T cells co‐cultured with 786‐O cells were derived from 5 healthy adult donors aged 25–35 years. The inclusion criteria of donors were no autoimmune diseases, no history of infection or drug treatment in the past 3 months, and those with malignant tumors and immunodeficiency were excluded. A volume of 20 mL peripheral venous blood was collected from each donor, and PBMCs were isolated by Ficoll–Hypaque density gradient centrifugation. CD8+ T cells were purified from PBMC by magnetic cell sorting kit (Miltenyi Biotec). Seventy‐two hours later, CD8^+^ T cells were harvested, stained with anti‐CD8‐PerCP and antigranzyme B‐APC antibodies, and evaluated for T‐cell activation marker expression by flow cytometry.

#### 2.11.4. Flow Cytometry Gating Strategy

(1) CD8+ T cells: forward scatter (FSC)‐A/side scatter (SSC)‐A ⟶ single cells (FSC‐H/FSC‐A) ⟶ live cells (PI−) ⟶ CD3+ ⟶ CD8+ ⟶ granzyme B+. (2) Intratumoral immune cells: FSC‐A/SSC‐A ⟶ single cells ⟶ live cells ⟶ CD45+ ⟶ CD3+/CD11b+ ⟶ subpopulations (CD8+, CD4+, Tregs, macrophages).

### 2.12. Animal Material

Eighteen male C57BL/6 nude mice aged 6–8 weeks were obtained from Beijing Weitong Lihua Laboratory Animal Technology Co., Ltd. and housed under specific pathogen‐free (SPF) conditions for 1 week prior to experimentation to allow acclimatization. Approved by the Animal Ethics Committee of Bengbu Medical University (Approval number: 2024‐No. 378). Before inoculation, all mice were clinically healthy, active, and free of visible abnormalities, with comparable baseline body weights across groups (18–23 g).

### 2.13. Subcutaneous Xenograft Model

Suspensions (200 μL) containing either normal cultured (blank group, *n* = 6), Silence‐METTL3 (silence‐METTL group, *n* = 6), or Silence‐METTL3 + 1,3‐diCQA‐intervened (Silence‐METTL3 786‐O cells pretreated with 1,3‐diCQA, *n* = 6) 780‐O cells (5 × 10^6^ cells) were subcutaneously inoculated into the right scapular region of each mouse. After 14 days, the mice were humanely euthanized (cervical dislocation method: lift the tail of the mouse and press the neck so that the cervical vertebra is dislocated and the spinal cord is broken, and the animal dies immediately), and tumors were carefully excised, photographed, and weighed. Tumor tissues were then processed for IHC analysis to evaluate the expression levels of PD‐L1 (1:200 dilution) and CD163 (1:200 dilution), following the same method as described above. Tumor volume was calculated as length × width^2^ × 0.5, where length represented the longest diameter and width the shortest diameter perpendicular to length. The maximum subcutaneous tumor volume allowed by the animal ethics committee of our hospital was 1500 mm^3^ or less.

### 2.14. Immunofluorescence Staining

Tumor sections were permeabilized using 0.5% Triton X‐100 and blocked with 5% BSA for 1 h. Primary antibodies of METTL3 (1:200), PI3K (1:100), and AKT (1:200) were added successively. After washing with PBS, the cells were incubated in the dark with secondary antibodies labeled with Cy3 (METTL3), FITC (PI3K), and Alexa Fluor 647 (AKT) for 1 h. Nuclei were counterstained with DAPI for 5 min, and after sealing with antifluorescence quench sealing agent, images were observed using confocal microscopy and acquired. Immunofluorescence images were acquired using a confocal microscope (Zeiss LSM 880) with a 40X objective lens. Scale bars (20 μm) are included in all IF images. Fluorescence intensity was quantified using ImageJ software, with mean fluorescence intensity of target proteins (METTL3, PI3K, and AKT) normalized to DAPI (nuclear marker) in 5 randomly selected fields per section.

### 2.15. Statistical Analysis

All data are presented as mean ± SD. Statistical analyses were performed using SPSS 25.0. Comparisons between two groups were performed using the independent‐samples *t* test, and comparisons among multiple groups were performed using one‐way ANOVA followed by the Bonferroni post hoc test. Homogeneity of variance was assessed before ANOVA, and the Bonferroni procedure was applied when the equal‐variance assumption was met. A *p* < 0.05 was considered statistically significant.

## 3. Results

### 3.1. METTL3 Expression in RCC

The analysis revealed significantly higher METTL3 protein expression in RCC tissues compared to adjacent tissues (*p* < 0.05, Figure 1(a)). Consistent with this finding, IHC staining demonstrated a markedly increased METTL3‐positive rate in tumor tissues was as high as [(52.95 ± 4.83)%] (*p* < 0.05, Figure 1(b)). In the pan‐cancer analysis, there was no significant differential expression at the transcript level in TCGA‐KIRC (Figure 1(c)), and no significant association with prognosis in KIRC (Figure 1(d)). Immune infiltration analysis via IOBR revealed that METTL3 expression was not correlated with the global immune infiltration score (a composite index integrating multiple immune cell subsets, Figure 1(e)), but was specifically positively correlated with CD4+ T cells and neutrophils in TCGA‐KIRC (Figure 1(f)). This subtype‐specific correlation is biologically meaningful, as CD4+ T cells in RCC often exhibit an exhausted phenotype (high PD‐1 expression), and neutrophils secrete proinflammatory cytokines (e.g., IL‐6, TNF‐α) to promote immunosuppression and tumor progression—aligning with our finding that METTL3 promotes immune evasion by upregulating PD‐L1 and impairing CD8+ T‐cell function. Although IL‐6 and TNF‐α were not directly quantified in the current cohort, the positive association between METTL3 expression and neutrophil infiltration supports a potential link between METTL3 and a cytokine‐rich inflammatory microenvironment in RCC.

FIGURE 1Expression of METTL3 in RCC. (a) Western blot detection of METTL3 protein expression in tissues. (b) IHC staining to detect METTL3 positivity in tissues. Scale bar = 20 μm (high‐power field, 400×). (c) Biological information analysis of METTL3 expression in various tumors. (d) Biological information analysis of the relationship between METTL3 and prognosis of various tumors. (e) Biological information analysis of the relationship between METTL3 and the degree of immune infiltration of renal tumors. (f) Biological information analysis of the relationship between METTL3 and kidney tumor immune cells. Abbreviations: ACC, adrenocortical carcinoma; KICH, kidney chromophobe; KIPAN, pan‐kidney cohort; KIRC, kidney renal clear cell carcinoma; KIRP, kidney renal papillary cell carcinoma. ^∗^ *p* < 0.05.

**Figure:**
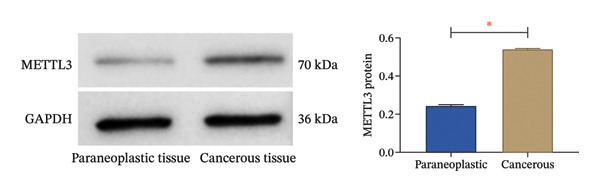
(a)

**Figure:**
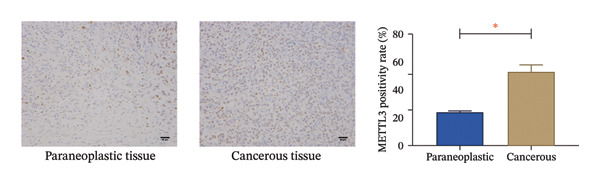
(b)

**Figure:**
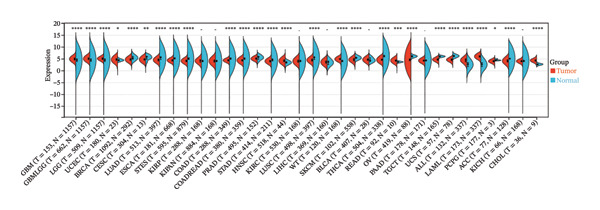
(c)

**Figure:**
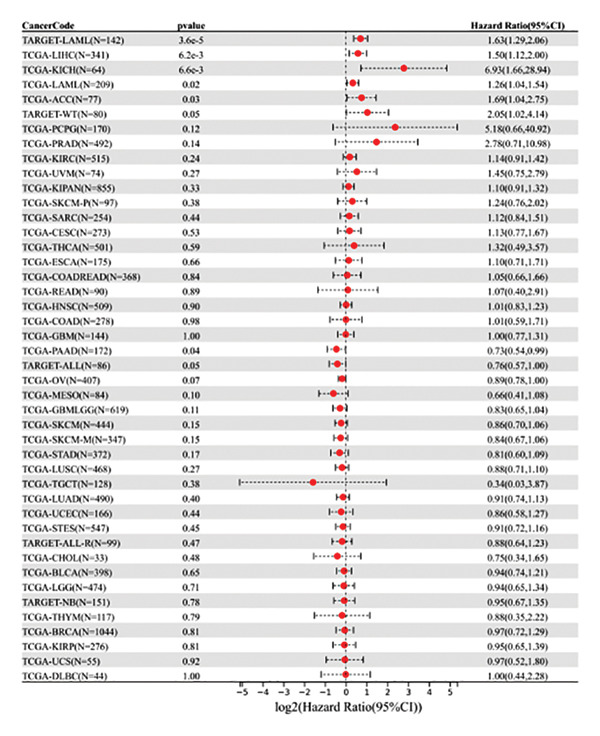
(d)

**Figure:**
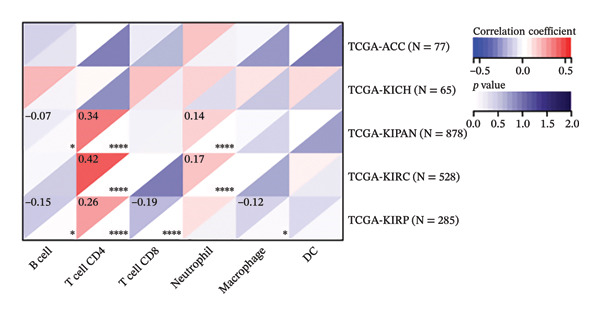
(e)

**Figure:**
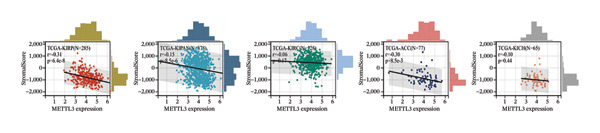
(f)

### 3.2. METTL3 Modulates 786‐O Cell Activity via the PI3K/AKT Pathway

Following intervention, METTL3 protein expression was significantly reduced in the Silence‐METTL3 group, restored to a level comparable to the blank group in the Silence‐METTL3 + 1,3‐diCQA group, and markedly increased in the Elevated‐METTL3 group (*p* < 0.05), confirming successful modulation of METTL3 expression. Notably, the phosphorylation ratios of PI3K (p‐PI3K/PI3K) and AKT (p‐AKT/AKT) were diminished upon METTL3 knockdown but enhanced with METTL3 overexpression (*p* < 0.05), demonstrating that METTL3 upregulation activates the PI3K/AKT pathway. Consistent with this finding, 1,3‐diCQA treatment similarly increased p‐PI3K/PI3K and p‐AKT/AKT expression (*p* < 0.05), confirming pathway activation. However, the Silence‐METTL3 + 1,3‐diCQA group showed phosphorylation levels comparable to the blank group (*p* > 0.05, Figure 2(a)). In addition, detecting the expression of m6A in each group of cells showed that increasing the expression of METTL3 could also activate the level of m6A. However, silencing METTL3 had the opposite effect (*p* < 0.05, Figure 2(b)). In biological behavior assays, cell proliferation and invasion in the Silence‐METTL3 group decreased compared to the blank group, while the apoptosis rate increased; conversely, the Elevated‐METTL3 group showed the opposite trends (*p* < 0.05), indicating that high METTL3 expression promotes the growth of 786‐O cells. Furthermore, cell activity in the 1,3‐diCQA group increased compared to the blank group (*p* < 0.05), whereas the Silence‐METTL3 + 1,3‐diCQA group showed no difference from the blank group (*p* > 0.05, Figures 2(c)–2(e)). Finally, using a METTL3 enzyme activity dependence assay, we observed that WT METTL3 restored PI3K/AKT pathway phosphorylation (vs. empty vector, *p* < 0.05), while the METTL3‐D395A mutant failed to rescue the phosphorylation level (vs. empty vector, *p* > 0.05, Figure 2(f)). Similarly, after silencing the m6A reading protein YTHDF1, the expression of p‐PI3K and p‐Akt was also observed (*p* < 0.05, Figure 2(g)).

FIGURE 2Effect of METTL3 on 786‐O. (a) Detection of METTL3 and PI3K/AKT pathway expression after interference with METTL3 and PI3K/AKT pathway. (b) The expression of m6A RNA in each group was detected. (c) Effect of METTL3 on the proliferative ability of 786‐O. Scale bar = 20 μm (high‐power field, 400×). (d) Effect of METTL3 on 786‐O invasion ability. (e) Effect of METTL3 on 786‐O apoptotic ability. (f) METTL3 enzyme activity dependence assay. (g) The changes of the PI3K/AKT pathway were observed by interfering with the m6A reading protein YTHDF1. ^∗^ indicates *p* < 0.05 compared to the blank group or empty vector. Quantitative data are presented as mean ± SD from three independent experiments. Abbreviations: D395A, catalytic‐dead METTL3 mutant; EdU, 5‐ethynyl‐2′‐deoxyuridine; Elevated‐METTL3, METTL3 overexpression group; Silence‐METTL3, METTL3 knockdown group; ns, not significant; WT, wild‐type METTL3.

**Figure:**
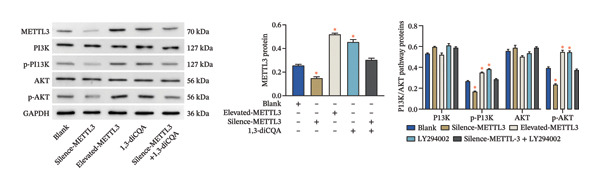
(a)

**Figure:**
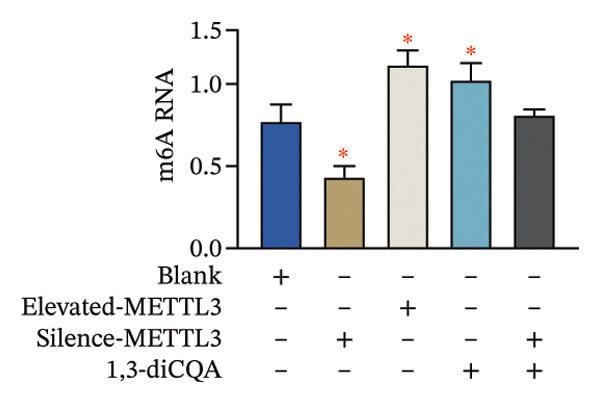
(b)

**Figure:**
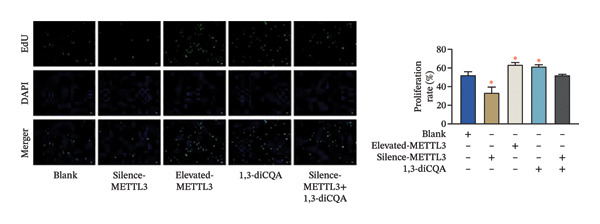
(c)

**Figure:**
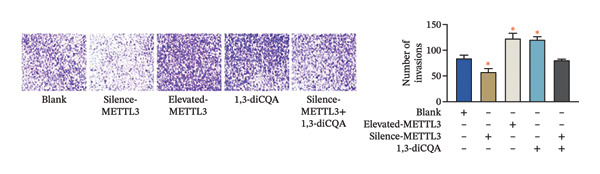
(d)

**Figure:**
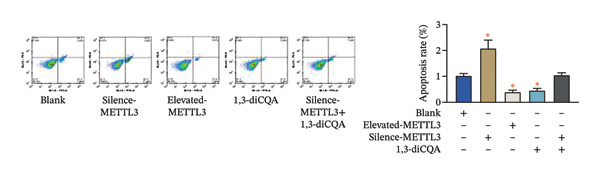
(e)

**Figure:**
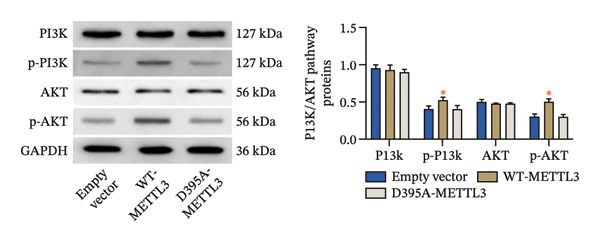
(f)

**Figure:**
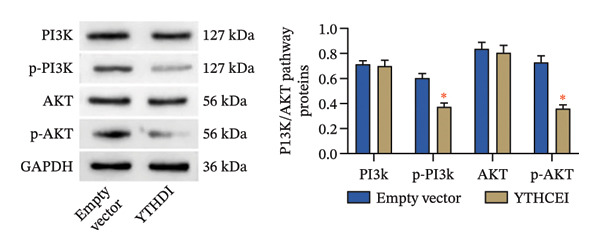
(g)

### 3.3. METTL3 Regulates the Immune Microenvironment of 786‐O Cells Through the PI3K/AKT Pathway

**FIGURE 3:**
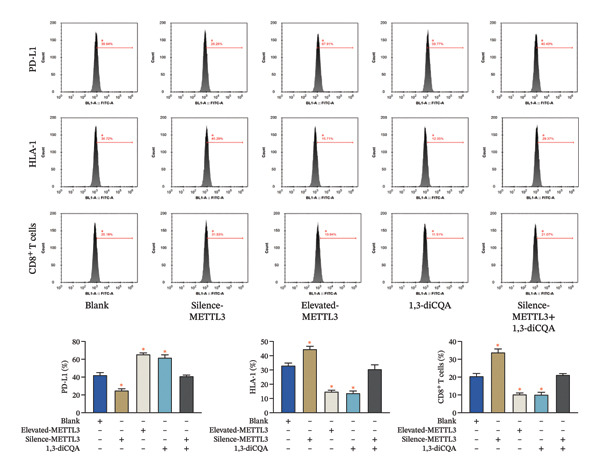
Detection of PD‐L1, HLA‐I, and CD8^+^ T‐cell expression to analyze the regulation of the 786‐O immune microenvironment by METTL3 through the PI3K/AKT pathway. ^∗^ indicates *p* < 0.05 compared to the blank group. Quantitative data are presented as mean ± SD from three independent experiments. Abbreviations: HLA‐I, human leukocyte antigen class I; PD‐L1, programmed death‐ligand 1.

Compared to the blank group, the Elevated‐METTL3 group exhibited significantly upregulated PD‐L1 expression along with downregulated HLA‐I levels and reduced CD8^+^ T cells (*p* < 0.05). Conversely, the Silence‐METTL3 group showed the opposite effects (*p* < 0.05), indicating that high METTL3 expression promotes immune evasion in 786‐O cells. A similar trend was observed in the 1,3‐diCQA group, which displayed elevated PD‐L1 and suppressed HLA‐I and CD8^+^ T cells (*p* < 0.05). In contrast, no significant differences in PD‐L1, HLA‐I, or CD8^+^ T cells were detected in the Silence‐METTL3 + 1,3‐diCQA group compared to the blank group (*p* > 0.05; Figure 3).

### 3.4. METTL3 Regulates RCC Growth via the PI3K/AKT Pathway

In this study, the volume of all subcutaneous tumors was within the limits specified by the ethics committee. Subcutaneous tumor growth analysis revealed no significant differences in tumor volume or weight between the Silence‐METTL3 + 1,3‐diCQA group and the blank group (*p* > 0.05). In contrast, the Silence‐METTL3 group showed a significant reduction in both parameters (*p* < 0.05; Figure 4(a)). Immunofluorescence staining showed that METTL3 was predominantly localized in the nucleus, while PI3K and AKT exhibited both nuclear and cytoplasmic localization in RCC tumor tissues (Figures 4(b)–4(d)). Consistent with these findings, IHC analysis indicated comparable PD‐L1 and CD163 positivity rates between the Silence‐METTL3 + 1,3‐diCQA group and the blank group (*p* > 0.05), whereas the Silence‐METTL3 group exhibited markedly decreased expression (*p* < 0.05; Figures 4(e) and 4(f)).

FIGURE 4Effect of METTL3 and growth of living tumors. (a) Weight and volume of subcutaneous tumors in mice. (b) Fluorescence staining to detect the expression and localization of METTL3. Scale bar = 20 μm (high‐power field, 400×). (c) Fluorescent staining to detect the expression and localization of PI3K. Scale bar = 20 μm (high‐power field, 400×). (d) Fluorescent staining to detect the expression and localization of AKT. Scale bar = 20 μm (high‐power field, 400×). (e) IHC staining to detect PD‐L1 positivity. (f) IHC staining to detect CD163 positivity. ^∗^ indicates *p* < 0.05 compared to the blank group. Quantitative data are presented as mean ± SD from three independent experiments. Abbreviations: IF, immunofluorescence; IHC, immunohistochemistry; ns, not significant.

**Figure:**
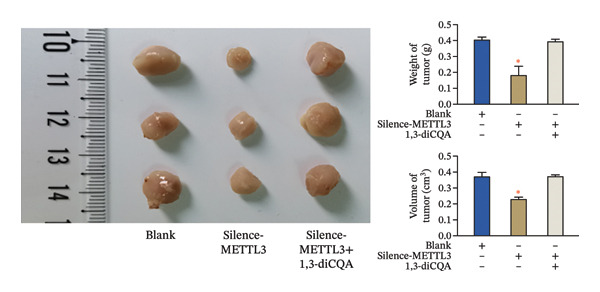
(a)

**Figure:**
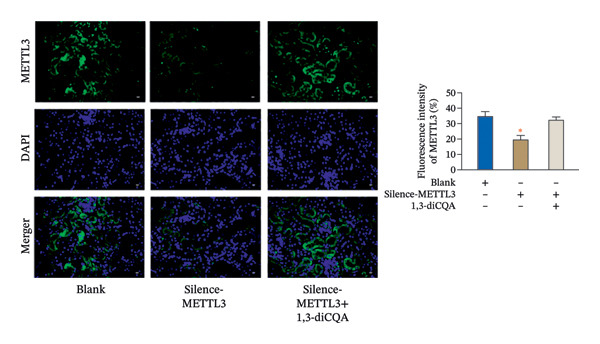
(b)

**Figure:**
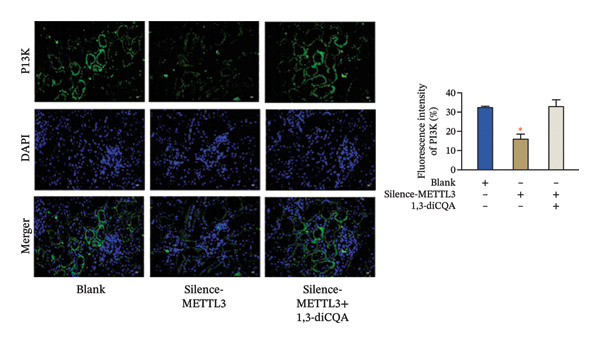
(c)

**Figure:**
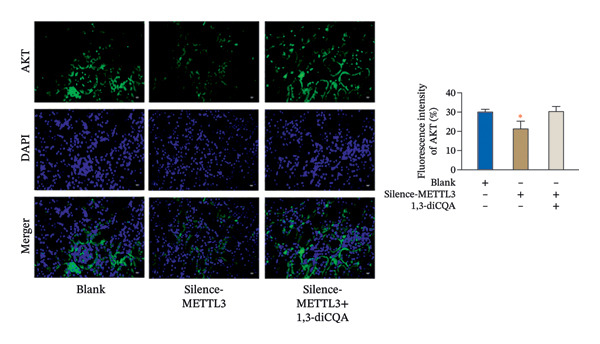
(d)

**Figure:**
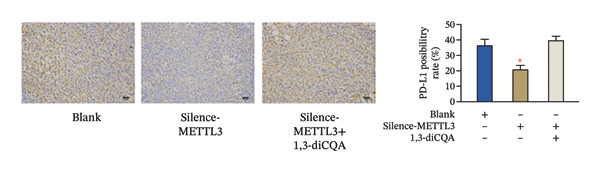
(e)

**Figure:**
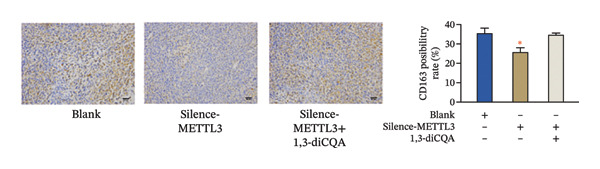
(f)

## 4. Discussion

This study found that METTL3 was upregulated in RCC. In addition, METTL3 promotes tumor cell proliferation, invasion, and immune evasion by activating the PI3K/AKT pathway, thereby reshaping the immunosuppressive tumor microenvironment. These findings highlight the METTL3–m6A–PI3K/AKT pathway axis as a critical regulatory hub in modulating the dynamic immune microenvironment of RCC.

First, IHC analysis of RCC tissues revealed elevated METTL3 expression, suggesting its potential role in RCC initiation and progression. However, the results of pan‐cancer analysis showed that METTL3 was not aberrantly expressed in KIRC. There may be three reasons for this: (1) Detection level differences: Our study quantified METTL3 protein expression (via Western blot and IHC), while TCGA‐KIRC data reflect mRNA levels—consistent with previous reports that m6A methyltransferase expression often exhibits mRNA–protein decoupling due to post‐transcriptional regulation [16]; (2) Clinical cohort heterogeneity: The 34 RCC patients in our study were all treatment‐naive, pathologically confirmed clear cell carcinoma (Fuhrman Grades 1–3, Stages I–II), whereas TCGA‐KIRC includes patients with diverse clinical characteristics (e.g., advanced stage, adjuvant therapy history, and mixed histologic subtypes); and (3) Sample quality variations: TCGA samples may include partially necrotic or stroma‐rich tissues, which could dilute tumor cell‐specific METTL3 signals. It is plausible that METTL3 upregulation is a relatively early event in RCC carcinogenesis, which may be masked in advanced tumors by complex adaptive mechanisms (e.g., immune checkpoint dysregulation or pathway crosstalk). Future studies with larger, stage‐matched cohorts are needed to confirm the clinical relevance of METTL3 protein expression in RCC. Immuno‐infiltration analysis via IOBR revealed that METTL3 expression was not correlated with the global immune infiltration score (sum of all immune cell subsets) but was specifically positively correlated with CD4+ T cells and neutrophils in TCGA‐KIRC—suggesting a subtype‐specific rather than global regulatory effect on immune infiltration. Given the established role of neutrophil‐derived inflammatory mediators such as IL‐6 and TNF‐α in RCC‐associated immune dysfunction, the observed association between METTL3 and neutrophil infiltration raises the possibility that the METTL3–PI3K/AKT axis may also contribute to cytokine‐driven inflammatory remodeling. Previous studies have shown that neutrophils in the RCC tumor microenvironment promote angiogenesis and immune suppression [17], while exhausted CD4+ T cells fail to support antitumor immunity and may even enhance immunosuppression [18]. Our findings indicate that METTL3 may shape the immunosuppressive microenvironment by enriching these pro‐tumor immune cells—consistent with the observed upregulation of PD‐L1 and downregulation of HLA‐I, which further impair antitumor T‐cell responses. The quantification of global m6A levels revealed that METTL3 overexpression significantly enhanced m6A modification (*p* < 0.05), whereas METTL3 silencing suppressed it, confirming METTL3’s catalytic function in installing m6A marks in RCC cells. This result aligns with the canonical role of METTL3 as the core methyltransferase and establishes a foundational link between METTL3 expression and m6A abundance in the RCC context. Subsequent cell experiments further confirmed that METTL3 overexpression enhanced the proliferative capacity of 786‐O cells while suppressing apoptosis, consistent with its established oncogenic function in various solid tumors. For instance, in hepatocellular carcinoma, METTL3 stabilizes oncogenic transcripts (e.g., MYC, EGFR) via m6A modification, thereby accelerating tumor progression [19]. Similarly, in colorectal cancer, METTL3 facilitates metastasis by activating the CXCL1/CXCR2 pathway [20]. Notably, silencing METTL3 significantly inhibited 786‐O cell proliferation and invasion, implying that METTL3 exerts its oncogenic effects through m6A‐dependent post‐transcriptional regulation of key oncogenes or signaling pathways. Moreover, METTL3 upregulation was associated with increased PD‐L1 expression, decreased HLA‐I levels, and impaired CD8^+^ T‐cell function. This indicates that METTL3 not only drives intrinsic tumor cell proliferation but also plays a pivotal role in remodeling the immune microenvironment to favor immune evasion.

Furthermore, this study provides evidence that METTL3 activates the PI3K/AKT pathway via an m6A‐dependent mechanism in RCC, positioning it as a regulator at the interface between tumor cell‐intrinsic behavior and the immune microenvironment [21, 22]. The PI3K/AKT pathway axis is constitutively activated in ∼40% of clear cell RCC, driving metabolic reprogramming (e.g., glycolysis) and anti‐apoptosis by phosphorylating downstream substrates such as FOXO1 [23, 24]. Our current study provides novel insights by demonstrating that PI3K/AKT pathway activation not only enhances the proliferative capacity of 786‐O cells but also facilitates immune evasion through dual mechanisms: upregulation of PD‐L1 expression and concurrent suppression of HLA‐I presentation. These findings align with and extend previous observations in other malignancies. For instance, in gastric cancer, PI3K/AKT pathway‐mediated STAT3 activation has been shown to upregulate PD‐L1, resulting in T‐cell exhaustion [25], while in liver cancer, AKT signaling impairs antigen presentation by downregulating critical components of the MHC class I processing machinery like TAP1 [26]. Particularly noteworthy is our discovery that PI3K/AKT pathway activation can partially counteract the amelioration of the immunosuppressive tumor microenvironment achieved through METTL3 silencing. This compelling evidence positions the PI3K/AKT pathway as a crucial downstream mediator of METTL3, orchestrating its dual regulatory effects on both tumor cell biology and immune microenvironment modulation. More importantly, the enzyme activity dependence assay demonstrated that the regulatory effects on PI3K/AKT phosphorylation require METTL3 methyltransferase activity. The WT‐METTL3 restored PI3K/AKT pathway activation in Silence‐METTL3 cells, whereas the catalytic dead mutant METTL3‐D395A failed to rescue the pathway activity. This critical experiment excludes the possibility of noncatalytic roles of METTL3 (e.g., scaffolding functions) and underscores that m6A modification is indispensable for PI3K/AKT pathway activation. Furthermore, the involvement of the m6A reader protein YTHDF1 adds another layer of mechanistic insight. Silencing YTHDF1 suppressed p‐PI3K and p‐AKT, indicating that the m6A marks deposited by METTL3 are recognized by reader proteins to facilitate the translation or stability of PI3K/AKT pathway components.

Furthermore, this study provides evidence supporting that METTL3 activates the PI3K/AKT pathway via an m6A‐dependent mechanism in RCC, positioning it as a critical regulator at the interface of tumor cell‐intrinsic properties and the immune microenvironment. Mechanistically, our findings support an m6A‐dependent regulatory axis linking METTL3 to PI3K/AKT pathway activation in RCC, which is mediated by METTL3’s methyltransferase activity and involves the m6A reader YTHDF1. First, the D395A‐METTL3 mutant (deficient in methyltransferase activity) failed to restore PI3K/AKT pathway phosphorylation, excluding noncatalytic roles of METTL3 and confirming that m6A modification is required for pathway activation. Second, silencing YTHDF1 (a key m6A reader that recognizes m6A‐modified transcripts to regulate stability or translation [27]) reduced PI3K/AKT pathway phosphorylation, indicating that YTHDF1 is a downstream mediator of METTL3‐m6A signaling. While direct m6A regulation of PI3K/AKT pathway‐related transcripts has been reported in other tumor contexts, candidate transcripts in RCC may include PIK3CA and AKT1; however, this possibility requires transcript‐level validation in our model [28]. Our study provides correlative evidence that this regulatory pattern may be conserved in RCC—supported by the coordinated changes in global m6A levels, PI3K/AKT pathway activation, and YTHDF1‐dependent pathway modulation. Collectively, these data indicate that METTL3 activates the PI3K/AKT pathway via an m6A‐dependent “writer‐reader” axis, though the specific target transcripts require future validation. Immunofluorescence staining showed that METTL3 was predominantly localized in the nucleus (consistent with its role as an RNA methyltransferase [29]), while PI3K and AKT exhibited both nuclear and cytoplasmic localization in RCC tumor tissues. This dual localization of the PI3K/AKT pathway is consistent with previous reports in RCC, where oncogenic stress (e.g., hypoxia, growth factor signaling) induces PI3K/AKT pathway nuclear translocation to regulate transcription of pro‐tumor genes (e.g., cyclins, PD‐L1) [30, 31]. Notably, METTL3 silencing reduced the total expression of PI3K and AKT but did not alter their subcellular distribution ratio. It is important to emphasize that the spatial co‐localization of METTL3, PI3K, and AKT reflects their distribution pattern but does not directly confirm functional interaction—this is supported by our pathway activation and rescue experiments, which demonstrate that METTL3 modulates PI3K/AKT pathway activity via its methyltransferase function. The dual cytoplasmic–nuclear localization of the PI3K/AKT pathway may reflect their multifunctional roles: The cytoplasmic PI3K/AKT pathway regulates classical pro‐survival signaling, while the nuclear PI3K/AKT pathway modulates the transcription of immune‐related genes (e.g., PD‐L1)—consistent with our finding that METTL3‐PI3K/AKT pathway activation alters PD‐L1 and HLA‐I expression. Importantly, the functional link between METTL3 and the PI3K/AKT pathway is not based on co‐localization but on (1) reciprocal changes in the PI3K/AKT pathway phosphorylation upon METTL3 manipulation; (2) failure of catalytic‐dead METTL3 to activate the PI3K/AKT pathway; (3) the rescue of METTL3 silencing effects by the PI3K/AKT pathway activator (1,3‐diCQA); and (4) YTHDF1‐dependent pathway modulation—all of which collectively support the regulatory axis. An additional implication of our findings is that the METTL3‐PI3K/AKT axis may intersect with cytokine‐driven inflammatory remodeling in RCC. PI3K/AKT signaling is closely linked to the transcriptional control of inflammatory mediators and immune‐regulatory molecules, and our observation that METTL3 expression was associated with neutrophil infiltration supports this possibility. Although inflammatory cytokines were not directly measured in the present study, it is biologically plausible that METTL3‐dependent PI3K/AKT activation may contribute to a pro‐tumor inflammatory milieu that reinforces immune escape [32].

This exploratory study provides preliminary evidence that METTL3 modulates RCC growth and immune evasion via the PI3K/AKT pathway. While these findings are correlative and limited by model systems, they lay the groundwork for future studies to validate METTL3 as a therapeutic target in combination with ICIs. First, elevated METTL3 expression or activation of the PI3K/AKT pathway may serve as predictive biomarkers for immunotherapy resistance, enabling more precise patient stratification. Secondly, silencing METTL3 expression or blocking the PI3K/AKT pathway may become a novel molecular therapeutic option for RCC. METTL3‐directed therapy is beginning to move from preclinical proof‐of‐concept toward early translational development; for example, STM2457 has shown antitumor activity in preclinical models. By contrast, therapeutic targeting of the PI3K/AKT pathway remains complicated by pathway feedback, adaptive resistance, and on‐target toxicity, suggesting that biomarker‐guided combination strategies may be more practical than single‐agent treatment. Furthermore, the development of m6A modification‐based epigenetic therapies, exemplified by agents such as STM2457 [33], represents a promising approach to reprogramming the immunosuppressive tumor microenvironment.

Despite the novel insights, this study has several limitations that should be noted: Although this study defines a METTL3–PI3K/AKT pathway regulatory framework in RCC, the downstream m6A‐modified transcripts remain unresolved. Future studies should apply MeRIP‐seq, ideally in combination with RNA‐seq and functional validation, to identify METTL3‐regulated inflammatory‐ and immune‐related target genes that mediate microenvironment remodeling. Additionally, the dynamic changes in immune cell subsets (e.g., Tregs, M2 macrophages) within animal models remain unresolved. Future studies could leverage single‐cell RNA sequencing to delineate the precise regulatory role of METTL3 in shaping the immune microenvironment. Cell line specificity: All in vitro experiments were conducted in the 786‐O clear cell RCC line; future validation in additional RCC cell lines (e.g., ACHN, Caki‐1) is needed to confirm the generalizability of the METTL3–PI3K/AKT pathway axis. Simplified immune models: The in vitro immune model focused on CD8+ T cells and key molecules (PD‐L1/HLA‐I), without including other immune cell subsets (e.g., Tregs, MDSCs) that contribute to TIME complexity. These limitations highlight the exploratory nature of the current work, and future studies will address these gaps to strengthen the clinical relevance of the findings. Future clinical validation should include larger, prospectively collected RCC cohorts spanning a broader range of pathological grades and clinical stages to better define the stage‐specific relevance and translational value of METTL3 expression.

## 5. Conclusion

This exploratory study demonstrates that METTL3 promotes RCC growth (in vitro and in vivo) and modulates RCC‐CD8+ T‐cell crosstalk (in vitro) by activating the PI3K/AKT pathway. This effect requires METTL3’s methyltransferase activity and involves YTHDF1. Further validation in larger cohorts and more complex models is needed to confirm the clinical relevance of the METTL3–PI3K/AKT pathway axis in RCC immunotherapy.

## Author Contributions

Tao Cheng and Mingli Gu conceived and designed the project, Tao Cheng and Weiqiang Xu wrote and revised the manuscript, Zeyu Zha generated the data, Likai Mao analyzed the data, Jing Ning visualized the data, and Shengtong Wang and Xinjie Huang supervised the study.

## Funding

This study was supported by the Key Natural Science Project of Bengbu Medical University (no. 2023byzd109); Bengbu Science and Technology Innovation Guidance Project (no. 2024ZD0027).

## Disclosure

All authors gave final approval of the version to be published and agreed to be accountable for all aspects of the work.

## Ethics Statement

All the experimental procedures involving animals were conducted in accordance with ARRIVE guidelines and approved by the Animal Ethics Committee of Bengbu Medical University (Approval number: 2024‐No. 378). The study involving human subjects complied with the Declaration of Helsinki and was approved by the ethical committee of Bengbu Medical College (Approval number: 2024‐No. 163), and all participants provided written informed consent.

## Consent

Please see the ethics statement.

## Conflicts of Interest

The authors declare no conflicts of interest.

## Data Availability

Original data in this study are available from the corresponding author on reasonable requests.
